# EAT Thickness and BAT-Related Thermogenesis: Dual Imaging Phenotypes Associated with Hepatic Steatosis Severity in MASLD

**DOI:** 10.3390/diagnostics16172696

**Published:** 2026-08-24

**Authors:** Xing Hu, Tieying Zhang, Xuhui Zhang, Jing Han, Fang Wang, Yuan Zhang

**Affiliations:** 1 Center of Ultrasound and Functional Diagnosis, Beijing Youan Hospital, Capital Medical University, No. 8 Xitoutiao, Youanmenwai, Fengtai District, Beijing 100069, China; diandianer@126.com (X.H.);; 2 State Key Laboratory of Infrared Physics, Shanghai Institute of Technical Physics, Chinese Academy of Sciences, No. 500 Yutian Road, Hongkou District, Shanghai 200083, China

**Keywords:** metabolic dysfunction-associated steatotic liver disease, epicardial adipose tissue, brown adipose tissue, multimodal imaging phenotyping, controlled attenuation parameter, infrared thermography, hepatic steatosis

## Abstract

**Background:** Metabolic dysfunction-associated steatotic liver disease (MASLD) is associated with ectopic fat accumulation and alterations in adipose tissue function. However, the relationships of structural and thermogenic adipose imaging markers with hepatic steatosis remain incompletely understood. This study aimed to jointly evaluate the cross-sectional associations of epicardial adipose tissue (EAT) thickness and infrared thermography (IRT)-derived brown adipose tissue (BAT)-related thermogenic activity with controlled attenuation parameter (CAP)-defined hepatic steatosis severity in adults with suspected MASLD. **Methods:** In this cross-sectional study, 207 adults undergoing clinical evaluation for suspected MASLD underwent transient elastography to obtain the controlled attenuation parameter (CAP) for hepatic steatosis assessment. EAT thickness was measured by transthoracic echocardiography, and BAT-related thermogenic activity was assessed by infrared thermography using the supraclavicular-to-chest temperature difference (ΔTemp). Associations of these adipose imaging phenotypes with CAP were evaluated using correlation analyses, sequential multivariable linear regression models, and BAT-stratified analyses. **Results:** EAT thickness increased progressively across CAP-defined steatosis grades (*p* < 0.001) and was positively correlated with CAP (r = 0.637, *p* < 0.001), whereas ΔTemp decreased with increasing steatosis severity and was inversely correlated with CAP (ρ = −0.277, *p* < 0.001). In sequential multivariable regression models, EAT thickness remained independently associated with CAP across adjustments for age, sex, body mass index, metabolic variables, and ΔTemp (standardized β = 0.559–0.623; all *p* < 0.001). Both EAT thickness and ΔTemp were independently associated with CAP in the fully adjusted model, with a stronger association for EAT thickness (standardized β = 0.559 vs. −0.167; *p* < 0.001 and *p* = 0.004, respectively). Stratified analyses demonstrated consistent associations between EAT thickness and CAP across both BAT-low and BAT-high activity groups. **Conclusions:** Greater EAT thickness and lower IRT-derived ΔTemp were independently associated with greater CAP-defined hepatic steatosis severity, with EAT thickness showing the stronger standardized association. These complementary structural and thermogenic imaging correlates warrant prospective evaluation to clarify their directionality and clinical relevance.

## 1. Introduction

Metabolic dysfunction-associated steatotic liver disease (MASLD) is currently one of the most prevalent chronic liver diseases worldwide, affecting approximately 38% of the adult population, with its prevalence continuing to rise globally [1]. In addition to liver-related complications, including cirrhosis and liver-related mortality [2], MASLD is associated with extrahepatic outcomes, particularly cardiovascular disease and all-cause mortality [3,4]. Increasing evidence indicates that MASLD is not merely a liver-specific disorder, but a systemic metabolic disease characterized by ectopic fat accumulation, altered adipose tissue function, and chronic low-grade inflammation [4,5]. Imaging markers derived from different adipose compartments may provide complementary information on the metabolic features associated with MASLD. However, the relationships of structurally and functionally distinct adipose imaging measures with hepatic steatosis severity remain incompletely defined.

Epicardial adipose tissue (EAT) has emerged as an imaging marker of regional ectopic adiposity in MASLD. As a highly metabolically active visceral fat depot located between the myocardium and visceral pericardium, EAT exerts local and systemic metabolic effects through the secretion of pro-inflammatory and profibrotic mediators [6,7]. Increased EAT thickness has been associated with insulin resistance, hepatic steatosis, liver fibrosis, and adverse cardiometabolic outcomes [8,9], supporting its relevance as an imaging marker of regional ectopic fat accumulation.

Complementary to EAT, brown adipose tissue (BAT) represents a metabolically protective imaging phenotype associated with adaptive thermogenesis and energy homeostasis [10,11]. Reduced BAT activity has been linked to increased hepatic fat content, whereas greater BAT activity is associated with more favorable glucose and lipid metabolism and improved cardiometabolic health [12,13]. Although fluorine-18 fluorodeoxyglucose positron emission tomography/computed tomography (^18^F-FDG PET/CT) remains the reference standard for BAT assessment, its clinical application is limited by radiation exposure and cost. Magnetic resonance imaging (MRI) provides an alternative approach but remains constrained by limited accessibility and the lack of standardized protocols [14,15]. Infrared thermography (IRT) has recently emerged as a practical, noninvasive, and radiation-free technique for estimating BAT-related thermogenic activity, providing a feasible approach for BAT imaging phenotyping [16,17].

Controlled attenuation parameter (CAP), obtained by vibration-controlled transient elastography, is widely used for the noninvasive assessment of hepatic steatosis and has shown good diagnostic performance against liver biopsy. Nevertheless, CAP remains a surrogate measure with limited discrimination between adjacent grades and may be influenced by body mass index (BMI), diabetes status, probe type, and liver disease etiology [18,19]. EAT thickness and IRT-derived ΔTemp reflect distinct adipose-related imaging domains—regional ectopic fat accumulation and BAT-related thermogenic response, respectively. However, their joint associations with hepatic steatosis in MASLD remain incompletely understood. Therefore, this study aimed to jointly evaluate the cross-sectional associations of echocardiography-derived EAT thickness and IRT-derived ΔTemp with CAP-defined hepatic steatosis severity and to assess whether they represent complementary adipose imaging phenotypes in adults evaluated for suspected MASLD.

## 2. Materials and Methods

### 2.1. Study Population

This single-center cross-sectional study consecutively enrolled adults referred to Beijing Youan Hospital, Capital Medical University, for evaluation of suspected MASLD between August 2024 and December 2025. Suspicion was based on ultrasonographic hepatic steatosis, metabolic abnormalities, and/or elevated liver enzymes. MASLD was diagnosed from baseline clinical, laboratory, and imaging data according to the current multisociety Delphi criteria, requiring imaging-confirmed steatosis and at least one cardiometabolic risk factor [20].

The inclusion criteria were as follows: (1) age ≥ 18 years; (2) undergoing clinical evaluation for suspected MASLD with available FibroScan examination; (3) available echocardiographic measurement of EAT thickness; and (4) available IRT data for assessment of BAT-related thermogenic activity.

The exclusion criteria were as follows: (1) viral hepatitis, alcohol-related liver disease, autoimmune liver disease, inherited metabolic liver disease, or other established chronic liver diseases; (2) excessive alcohol consumption (≥30 g/day for men or ≥20 g/day for women); (3) previous liver surgery, malignancy, or severe infectious disease; (4) established cardiovascular disease, including coronary artery disease, heart failure, clinically significant arrhythmia, or severe valvular heart disease; (5) missing key clinical or imaging data; and (6) unreliable CAP measurement according to the prespecified quality criteria.

The study protocol was approved by the Ethics Committee of Beijing Youan Hospital, Capital Medical University (Approval No. LL-2024-074-K). Written informed consent was obtained from all participants prior to enrollment.

### 2.2. Clinical and Laboratory Assessments

Demographic, anthropometric, and laboratory data were collected for all participants. Demographic and anthropometric variables included age, sex, and BMI. Laboratory parameters included alanine aminotransferase (ALT), aspartate aminotransferase (AST), total cholesterol (TC), triglycerides (TG), low-density lipoprotein cholesterol (LDL-C), and high-density lipoprotein cholesterol (HDL-C).

### 2.3. Transient Elastography and CAP Assessment

All participants underwent transient elastography (TE) using the FibroScan^®^ system (Echosens, Paris, France) to obtain CAP values. Examinations were performed after at least 6 h of fasting, with participants in the supine position and the right arm in maximal abduction. The M or XL probe was selected according to the manufacturer’s recommendations. A minimum of 10 valid measurements was required for each examination. Results were considered reliable when the success rate was ≥60% and the interquartile range-to-median ratio (IQR/M) was ≤30%. Steatosis grades were defined using biopsy-referenced CAP thresholds derived from an Asian cohort with chronic liver disease: S0 (<250 dB/m), S1 (250–298 dB/m), S2 (299–326 dB/m), and S3 (≥327 dB/m). In the original validation study, the AUROCs for detecting ≥S1, ≥S2, and S3 steatosis were 0.885, 0.894, and 0.800, respectively [18]. CAP was used to quantify hepatic steatosis severity rather than to establish the diagnosis of MASLD or determine study eligibility.

### 2.4. Echocardiographic Assessment of EAT Thickness

EAT thickness was measured by transthoracic two-dimensional echocardiography using an ACUSON SC2000 ultrasound system (Siemens Healthineers, Issaquah, WA, USA). Participants were examined in the left lateral decubitus position, and EAT was assessed in the standard parasternal long-axis view. EAT was defined as the echo-free space between the outer myocardial wall and the visceral pericardium. The maximum EAT thickness was measured perpendicularly over the free wall of the right ventricle at end-systole. The primary observer measured EAT thickness over three consecutive cardiac cycles, and the mean value was used for analysis (Figure S1) [21]. All echocardiographic examinations and EAT measurements were performed by experienced sonographers blinded to the CAP and IRT results. For reproducibility assessment, stored echocardiographic images from a subset of 20 participants were remeasured by the primary observer and independently evaluated by a second observer who was blinded to the primary observer’s measurements.

### 2.5. Infrared Thermography Assessment of BAT-Related Thermogenic Response

BAT-related thermogenic response was assessed by IRT using an InfiRay M600F infrared thermal camera (IRay Technology Co., Ltd., Yantai, China) [22,23,24]. The manufacturer-calibrated camera was checked before each examination. Examinations were performed in a temperature-controlled room maintained at 19 °C. Participants were instructed to avoid vigorous exercise, smoking, and caffeinated beverages for at least 12 h before the examination. No female participant underwent IRT examination during active menstruation.

Participants were seated with the neck and upper chest exposed, approximately 1 m from the camera, with skin emissivity set at 0.98. Baseline thermograms were acquired before participants underwent 30 min of standardized mild cold exposure at 19 °C. All participants completed the protocol without intolerance-related termination, and no overt shivering was documented. Post-exposure thermograms were acquired immediately thereafter.

On post-exposure thermograms, an evaluator blinded to CAP and EAT measurements manually delineated the regions of interest (ROIs) using dedicated software. The highest temperature within the bilateral supraclavicular ROIs was recorded as the supraclavicular skin temperature (Tscv), whereas the mean temperature within the parasternal upper chest reference ROIs was recorded as the chest reference temperature (Tc). Δ Temp was calculated as the post-exposure spatial temperature difference between these regions (ΔTemp = Tscv − Tc), with higher values indicating greater BAT-related thermogenic response (Figure S2).

### 2.6. Statistical Analysis

Continuous variables are presented as mean ± standard deviation (SD) or median (interquartile range [IQR]), as appropriate, and categorical variables as frequencies (percentages). Comparisons among CAP-defined steatosis grades were performed using one-way analysis of variance (ANOVA) or the Kruskal–Wallis test for continuous variables and the χ^2^ test for categorical variables, with post hoc comparisons performed using the Scheffé or Dunn–Bonferroni method, as appropriate.

Pearson or Spearman correlation analyses were performed, as appropriate, to assess the associations between EAT thickness, ΔTemp, and CAP. Trend analysis was conducted by entering CAP-defined steatosis grade as an ordinal variable in a linear regression model to assess the linear trend of EAT thickness across increasing steatosis severity.

Multivariable linear regression models were constructed with CAP as the dependent variable using sequential adjustment for sex, age, BMI, and metabolic variables (ALT, TG, and HDL-C). ΔTemp was analyzed as a continuous variable in all analyses. For exploratory stratified and interaction analyses, the sample median was used to obtain comparably sized BAT-low and BAT-high activity groups without outcome-driven cut-off selection; this operational cut-off should not be interpreted as a biological boundary or diagnostic threshold. Multicollinearity was assessed using variance inflation factors (VIFs) and tolerance values, with VIF ≥ 5 or tolerance ≤ 0.20 indicating potential concern. Intraobserver repeatability and interobserver reproducibility were quantified using intraclass correlation coefficients (ICCs) with 95% confidence intervals (CIs).

All statistical tests were two-sided, and a *p* value < 0.05 was considered statistically significant. Statistical analyses were performed using IBM SPSS Statistics version 25.0 (IBM Corp., Armonk, NY, USA).

## 3. Results

### 3.1. Participant Flow and Baseline Characteristics

**Figure 1:**
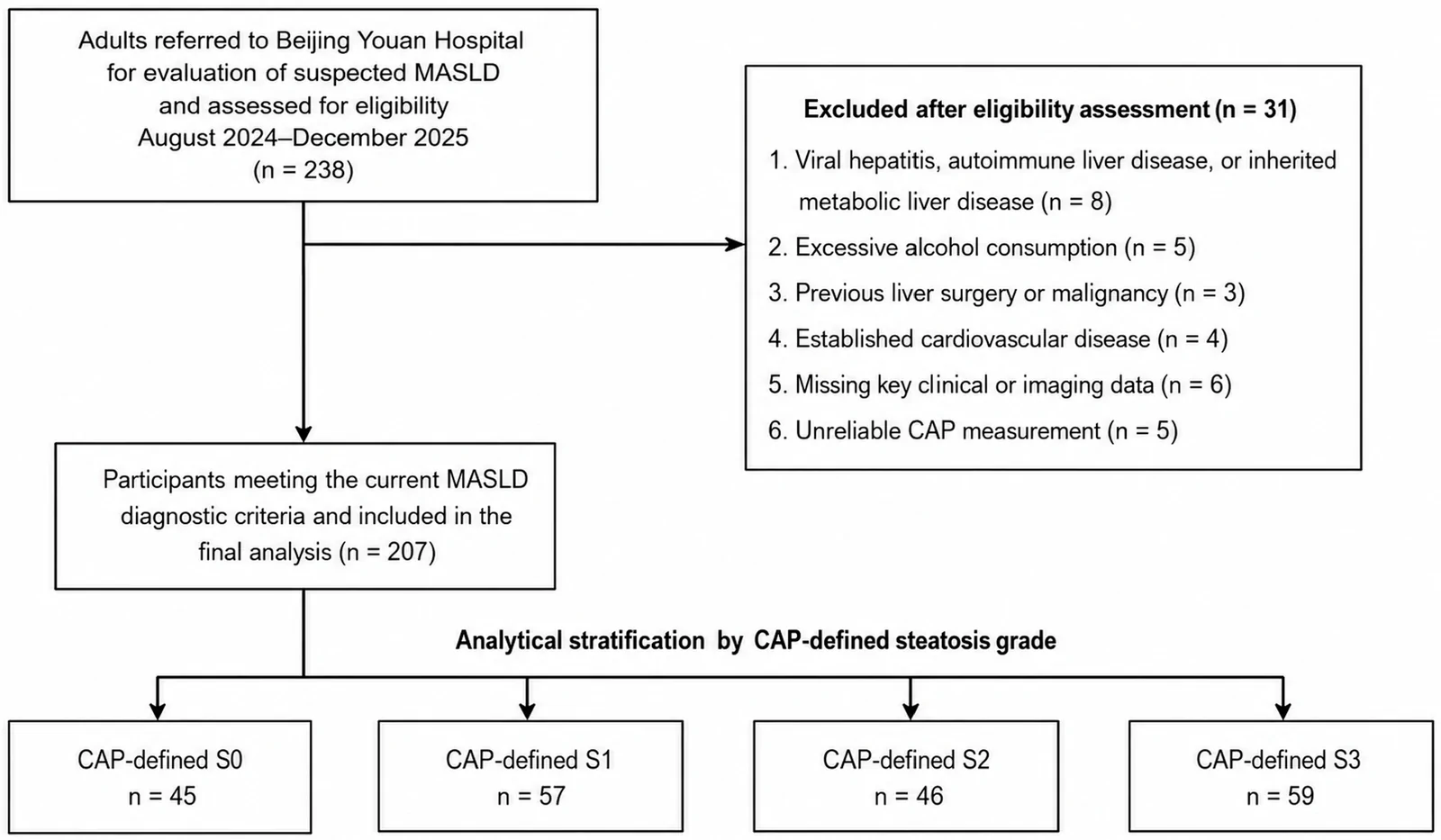
Participant flow diagram. Flow diagram showing participant assessment, exclusions with reasons, inclusion in the final analysis, and stratification by CAP-defined hepatic steatosis grade. Abbreviations: CAP, controlled attenuation parameter; MASLD, metabolic dysfunction-associated steatotic liver disease.

Of 238 adults assessed for eligibility, 31 were excluded for the reasons summarized in Figure 1, leaving 207 participants for the final analysis. All included participants met the current MASLD diagnostic criteria. For the study analyses, they were subsequently stratified into four CAP-defined categories: S0 (*n* = 45), S1 (*n* = 57), S2 (*n* = 46), and S3 (*n* = 59) (Table 1). Across increasing CAP-defined categories, the proportion of males and BMI progressively increased, whereas age decreased (all *p* < 0.001). Hypertension and diabetes mellitus were present in 48 (23.2%) and 46 (22.2%) participants, respectively, and neither condition differed significantly in prevalence across the four categories (*p* = 0.415 and *p* = 0.281, respectively). None of the included participants had a documented history of thyroid disease. ALT and AST differed significantly among groups (*p* = 0.005 and *p* = 0.002, respectively). Among lipid parameters, TC, TG, and LDL-C were comparable across groups, whereas HDL-C decreased with increasing steatosis severity (*p* = 0.019).

### 3.2. Association Between EAT and Hepatic Steatosis Severity

**Figure 2:**
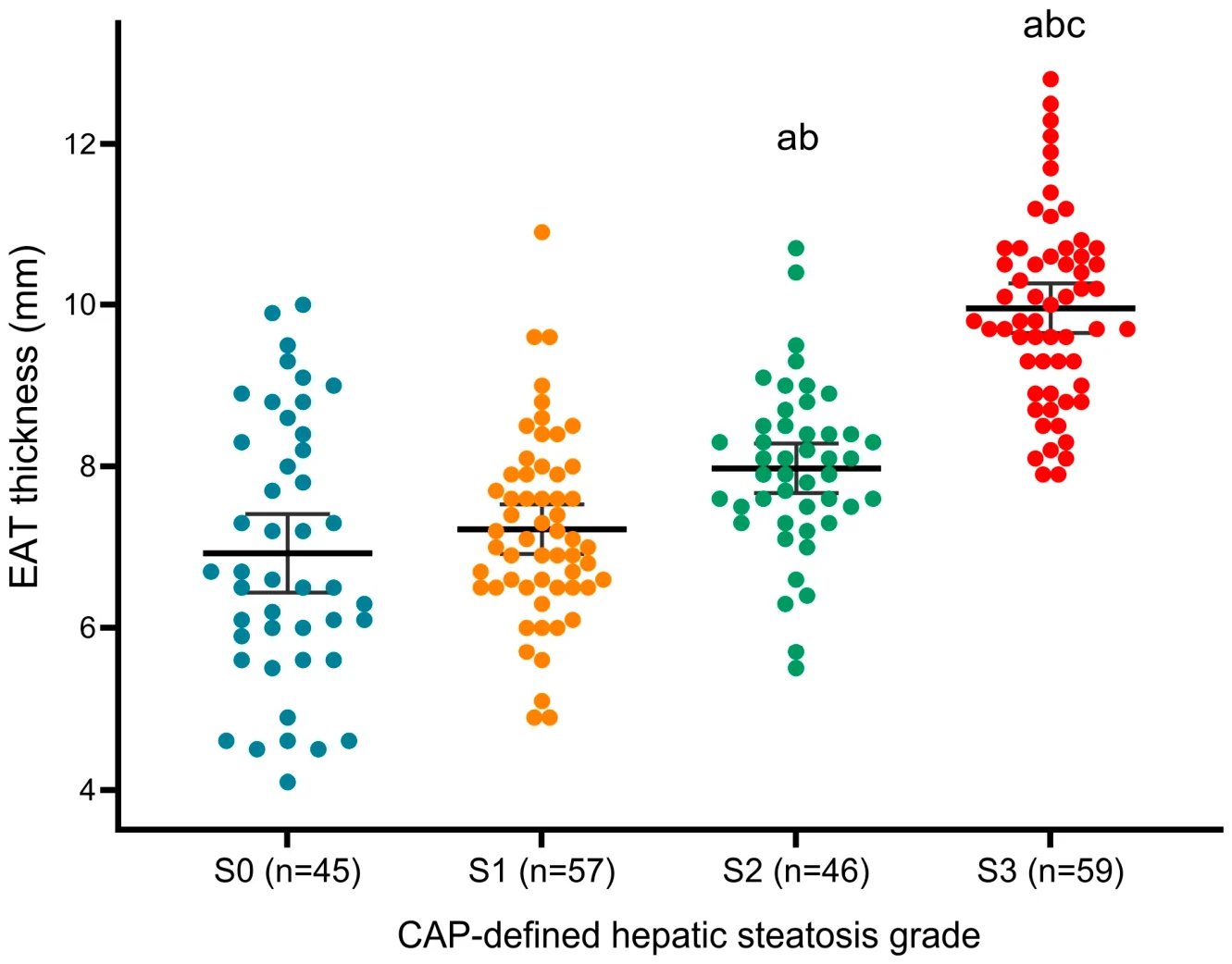
Epicardial adipose tissue thickness according to CAP-defined hepatic steatosis grade. Epicardial adipose tissue (EAT) thickness increased progressively with increasing CAP-defined hepatic steatosis severity. Individual data points are shown for each participant; horizontal lines indicate group means, and vertical error bars represent 95% confidence intervals (CIs). Superscript letters indicate statistically significant differences according to Scheffé post hoc comparisons: ^a^ *p* < 0.05 vs. S0, ^b^ *p* < 0.05 vs. S1, and ^c^ *p* < 0.05 vs. S2. Abbreviations: CAP, controlled attenuation parameter; CI, confidence interval; EAT, epicardial adipose tissue.

The distribution of EAT thickness across CAP-defined steatosis grades is shown in Figure 2. EAT thickness increased progressively with steatosis severity, with mean values of 6.9 ± 1.6 mm, 7.2 ± 1.2 mm, 8.0 ± 1.0 mm, and 10.0 ± 1.2 mm in the S0, S1, S2, and S3 groups, respectively (*p* < 0.001). Post hoc Scheffé analysis showed that EAT thickness was significantly greater in the S2 and S3 groups than in the S0 and S1 groups (all *p* < 0.05). In addition, the S3 group exhibited higher EAT thickness than the S2 group (*p* < 0.001), whereas no significant difference was observed between the S0 and S1 groups (*p* = 0.706). In the subset of 20 participants, EAT thickness measurements showed excellent intraobserver repeatability (ICC = 0.988, 95% CI: 0.970–0.995) and interobserver reproducibility (ICC = 0.960, 95% CI: 0.903–0.984).

**Figure 3:**
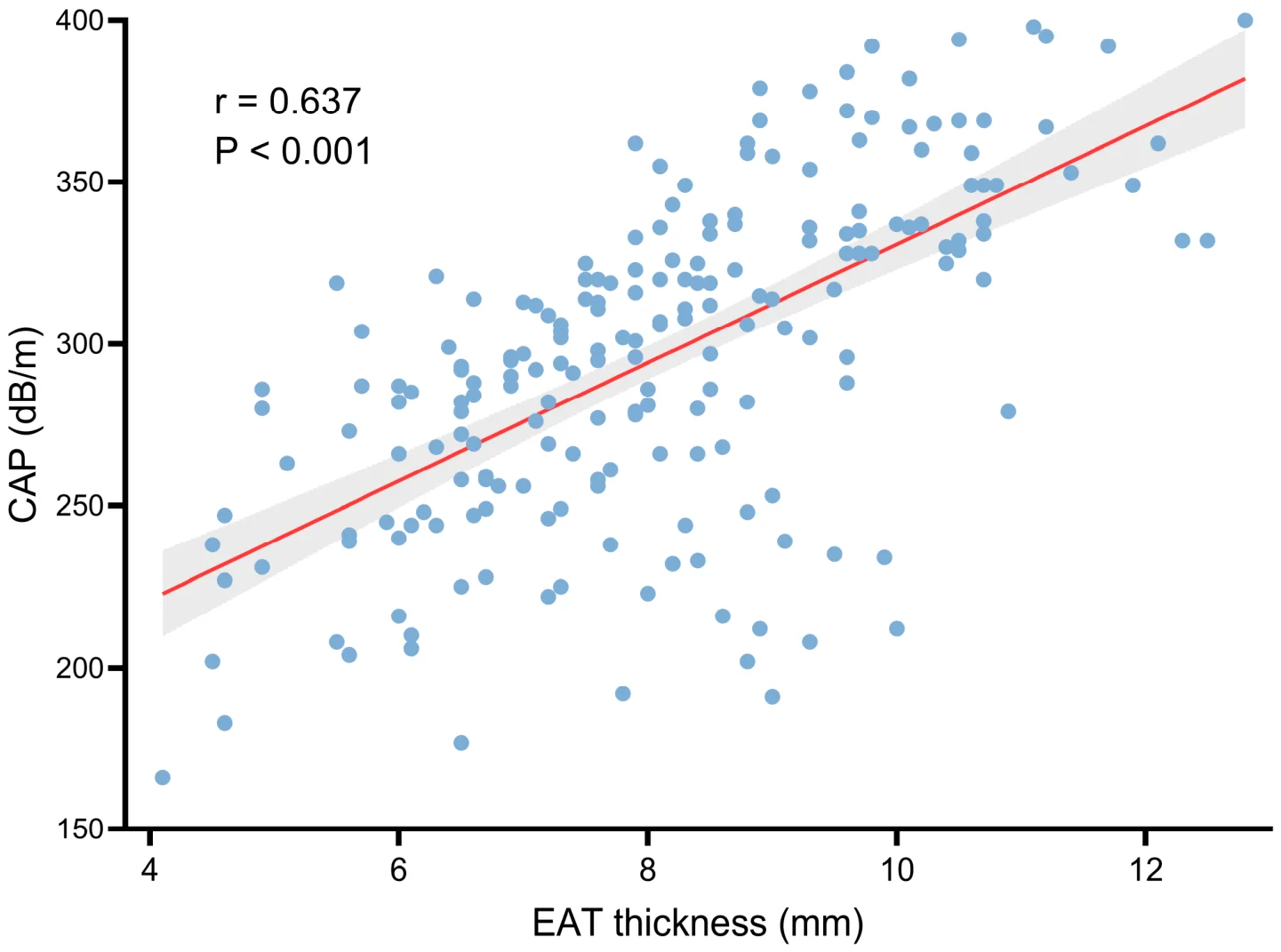
Association between epicardial adipose tissue thickness and controlled attenuation parameter. The scatter plot and fitted line depict the unadjusted bivariate relationship between epicardial adipose tissue (EAT) thickness and controlled attenuation parameter (CAP). The solid line represents the fitted linear regression line, and the shaded area indicates the 95% confidence interval (CI). Pearson correlation analysis showed a significant positive correlation between EAT thickness and CAP (r = 0.637, *p* < 0.001). In the fully adjusted model (Model 4), EAT thickness remained positively associated with CAP after adjustment for sex, age, BMI, ALT, TG, HDL-C, and IRT-derived ΔTemp (standardized β = 0.559, *p* < 0.001). Complete regression results are presented in Table 2. Abbreviations: ALT, alanine aminotransferase; BMI, body mass index; CAP, controlled attenuation parameter; CI, confidence interval; EAT, epicardial adipose tissue; HDL-C, high-density lipoprotein cholesterol; IRT, infrared thermography; TG, triglycerides.

Pearson correlation analysis demonstrated a significant positive correlation between EAT thickness and CAP (r = 0.637, *p* < 0.001) (Figure 3). Trend analysis further showed a significant linear increase in EAT thickness across steatosis grades (standardized β = 0.660, *p* for trend < 0.001). In linear regression analysis, each one-grade increase in steatosis severity was associated with a 1.03 mm increase in EAT thickness (95% CI: 0.869–1.192).

### 3.3. Association Between BAT-Related Thermogenic Activity and Hepatic Steatosis Severity

**Figure 4:**
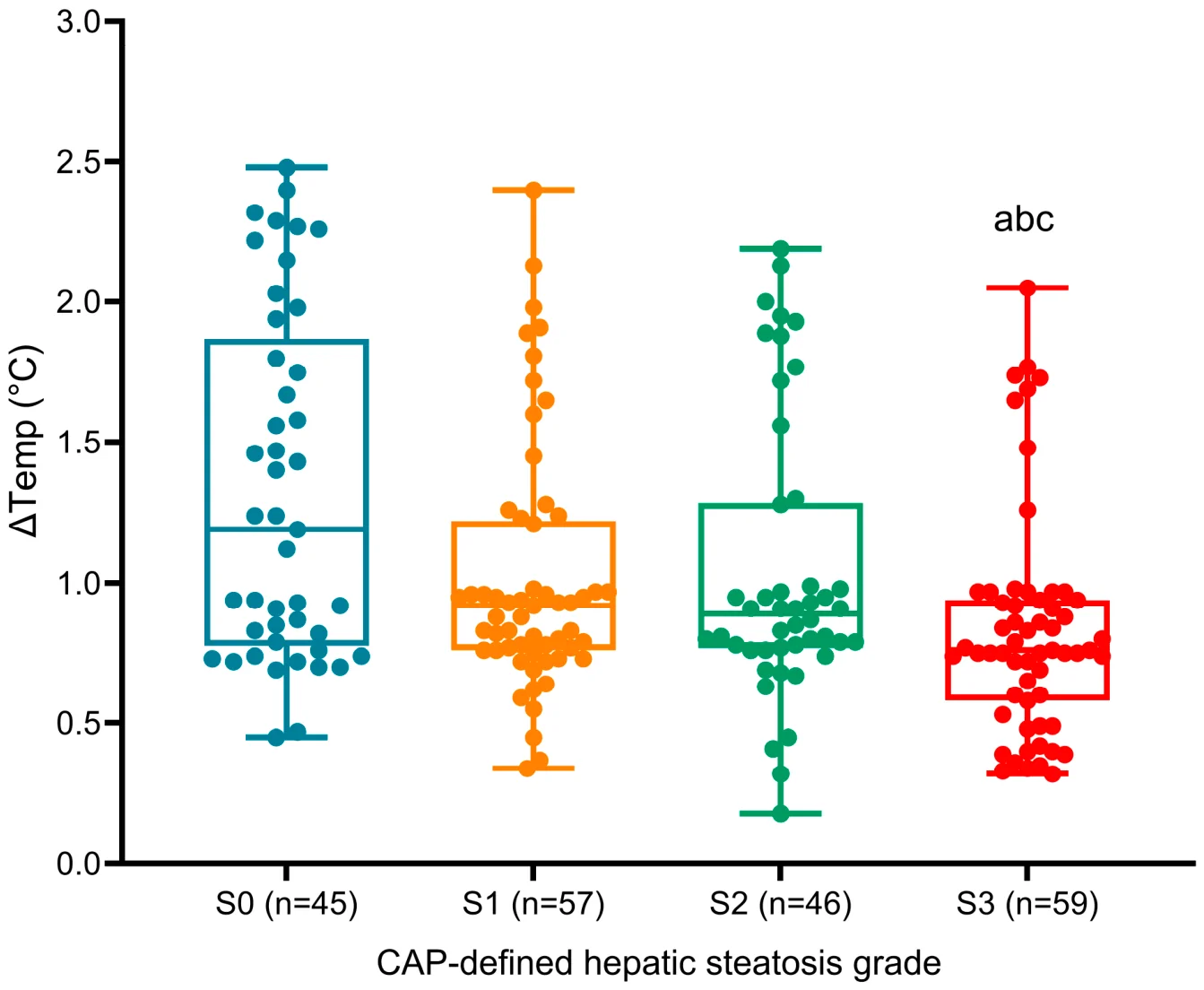
BAT-related thermogenic activity across CAP-defined hepatic steatosis grades. Box-and-whisker plots show BAT-related thermogenic activity (ΔTemp) across CAP-defined hepatic steatosis grades. The center line represents the median, the boxes indicate the interquartile range (IQR), and the whiskers denote the minimum and maximum values. Individual dots represent individual participants. Group differences were evaluated using the Kruskal–Wallis test followed by Dunn–Bonferroni post hoc comparisons. Superscript letters indicate statistically significant differences: ^a^ *p* < 0.05 vs. S0, ^b^ *p* < 0.05 vs. S1, and ^c^ *p* < 0.05 vs. S2. Abbreviations: BAT, brown adipose tissue; CAP, controlled attenuation parameter; ΔTemp, supraclavicular temperature minus chest reference temperature.

**Figure 5:**
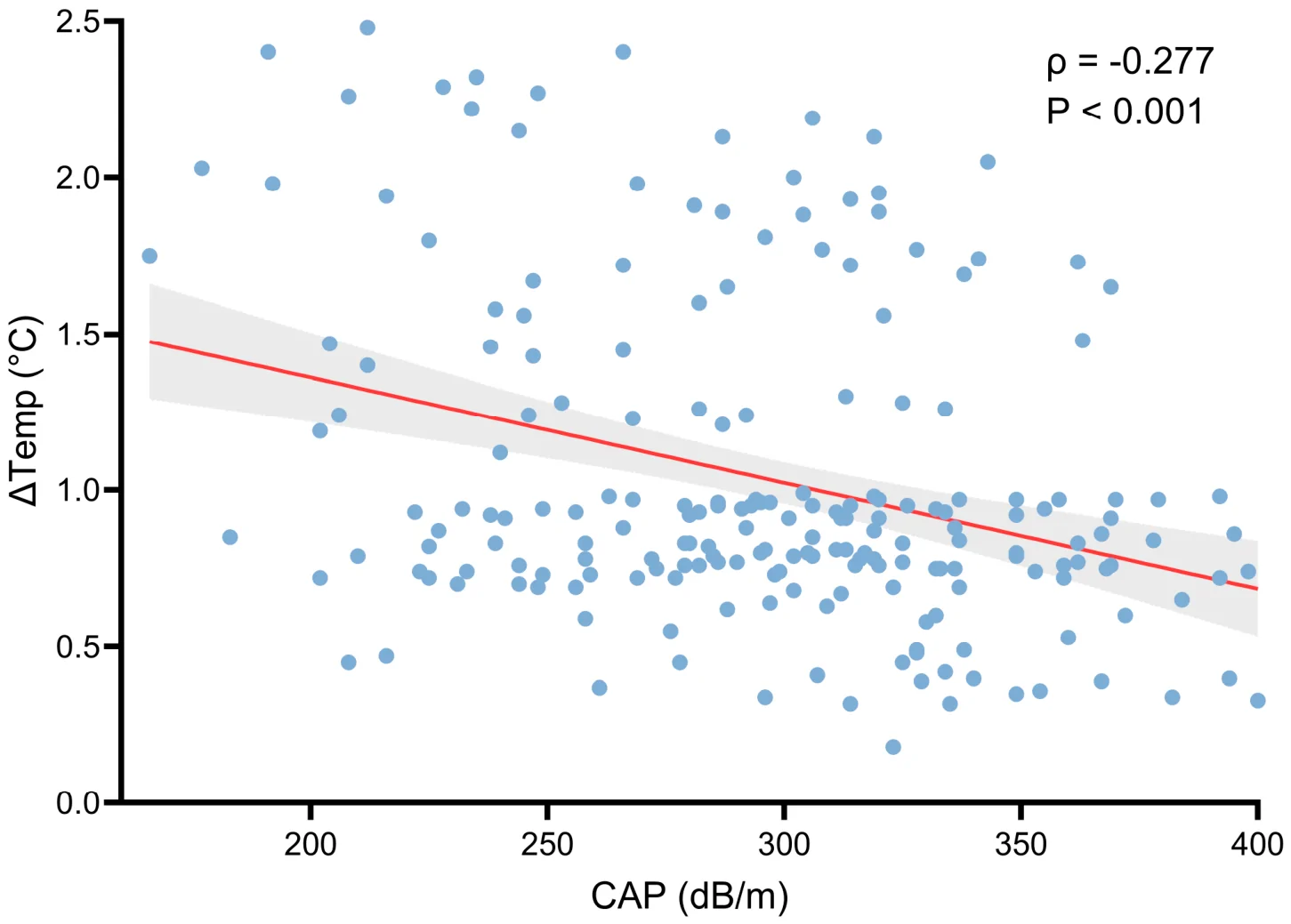
Association between BAT-related thermogenic activity and controlled attenuation parameter. The scatter plot and fitted line depict the unadjusted bivariate relationship between IRT-derived ΔTemp and controlled attenuation parameter (CAP). The solid line represents the fitted linear regression line, and the shaded area indicates the 95% confidence interval (CI). Spearman correlation analysis showed a significant inverse correlation between ΔTemp and CAP (ρ = −0.277, *p* < 0.001). In the fully adjusted model (Model 4), ΔTemp remained inversely associated with CAP after adjustment for sex, age, BMI, ALT, TG, HDL-C, and EAT thickness (standardized β = −0.167, *p* = 0.004). Complete regression results are presented in Table 2. Abbreviations: ALT, alanine aminotransferase; BMI, body mass index; CAP, controlled attenuation parameter; CI, confidence interval; EAT, epicardial adipose tissue; HDL-C, high-density lipoprotein cholesterol; IRT, infrared thermography; TG, triglycerides; ΔTemp, supraclavicular temperature minus chest reference temperature.

The distribution of ΔTemp across CAP-defined steatosis grades is shown in Figure 4. Kruskal–Wallis analysis demonstrated a significant difference in ΔTemp among groups (*p* < 0.001). ΔTemp progressively decreased with increasing steatosis severity, with median values of 1.19 (0.78–1.87) °C, 0.92 (0.76–1.22) °C, 0.89 (0.77–1.29) °C, and 0.76 (0.58–0.94) °C in the S0, S1, S2, and S3 groups, respectively. Post hoc Dunn–Bonferroni analysis showed that ΔTemp was significantly lower in the S3 group than in the S0, S1, and S2 groups (all *p* < 0.05), whereas no significant differences were observed among the S0, S1, and S2 groups. Spearman correlation analysis further demonstrated a significant inverse correlation between ΔTemp and CAP (ρ = −0.277, *p* < 0.001) (Figure 5).

### 3.4. Multivariable Linear Regression Analysis of CAP

To evaluate whether EAT and BAT-related thermogenic activity were independently associated with hepatic steatosis, multivariable linear regression models were constructed with CAP as the dependent variable using a sequential adjustment strategy (Table 2).

In Model 1, after adjustment for sex and age, EAT thickness was significantly associated with CAP (B = 17.422, standardized β = 0.597, *p* < 0.001). After further adjustment for BMI (Model 2), this association remained significant (B = 15.769, standardized β = 0.623, *p* < 0.001), with a larger standardized effect size than BMI (0.623 vs. 0.201).

Following additional adjustment for ALT, TG, and HDL-C (Model 3), the association between EAT thickness and CAP remained largely unchanged (B = 16.100, standardized β = 0.586, *p* < 0.001).

In the fully adjusted model (Model 4), which additionally included ΔTemp, EAT thickness remained independently associated with CAP (B = 15.371, standardized β = 0.559, *p* < 0.001). ΔTemp was also independently and inversely associated with CAP, although the magnitude of this association was more modest (B = −16.174, standardized β = −0.167, *p* = 0.004). Based on the absolute standardized coefficients, EAT thickness showed a substantially stronger association with CAP than ΔTemp (|β| = 0.559 vs. 0.167). BMI also remained significant (standardized β = 0.143, *p* = 0.022), although its effect size was smaller than that of EAT thickness. The standardized β coefficient for EAT thickness remained relatively stable across Models 1–4 (0.559–0.623). Additional adjustments for diabetes mellitus and hypertension yielded similar estimates for EAT thickness (standardized β = 0.551, *p* < 0.001) and ΔTemp (standardized β = −0.166, *p* = 0.004) (Table S1).

### 3.5. Stratified Regression Analysis According to BAT Activity

**Figure 6:**
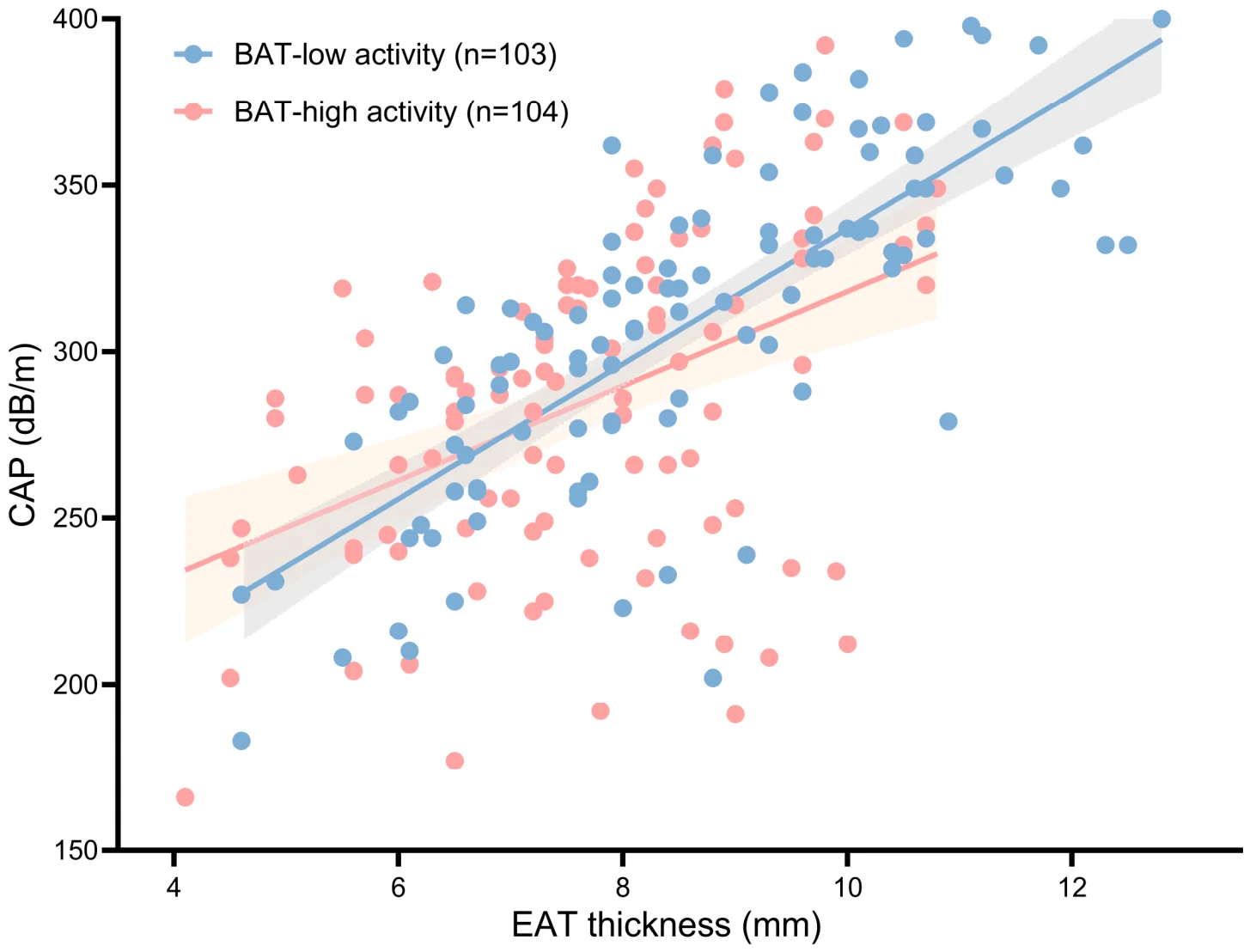
Association between epicardial adipose tissue thickness and controlled attenuation parameter stratified by BAT activity status. The scatter plots and fitted lines depict the unadjusted bivariate relationships between epicardial adipose tissue (EAT) thickness and controlled attenuation parameter (CAP) within the BAT-low (*n* = 103) and BAT-high (*n* = 104) groups, defined by the sample median ΔTemp. Solid lines represent fitted linear regression lines, and shaded areas indicate 95% confidence intervals (CIs). In separate multivariable models adjusted for sex, age, BMI, ALT, TG, HDL-C, and ΔTemp, EAT thickness remained positively associated with CAP in both the BAT-low group (B = 17.191, standardized β = 0.662, *p* < 0.001) and the BAT-high group (B = 15.140, standardized β = 0.511, *p* < 0.001). Complete regression results are presented in Table 3. The median-based groups reflect relative ΔTemp levels within this cohort and do not represent validated biological or diagnostic categories. Abbreviations: ALT, alanine aminotransferase; BAT, brown adipose tissue; BMI, body mass index; CAP, controlled attenuation parameter; CI, confidence interval; EAT, epicardial adipose tissue; HDL-C, high-density lipoprotein cholesterol; TG, triglycerides; ΔTemp, supraclavicular-to-chest temperature difference.

To further evaluate the relationship between EAT thickness and hepatic steatosis under different BAT activity states, participants were stratified according to the median ΔTemp value, and separate multivariable linear regression models were constructed (Table 3). The associations between EAT thickness and CAP in the BAT-low and BAT-high activity groups are illustrated in Figure 6.

In the BAT-low activity group, EAT thickness was independently associated with CAP (B = 17.191, standardized β = 0.662, *p* < 0.001). BMI (standardized β = 0.275, *p* < 0.001), male sex (standardized β = 0.216, *p* = 0.004), and ALT (standardized β = 0.138, *p* = 0.043) were also significantly associated with CAP, whereas ΔTemp was not (*p* = 0.694).

In the BAT-high activity group, EAT thickness remained independently associated with CAP (B = 15.140, standardized β = 0.511, *p* < 0.001). In addition, ΔTemp was inversely associated with CAP (B = −27.241, standardized β = −0.282, *p* = 0.006), whereas BMI was not significantly associated with CAP (*p* = 0.956). No statistically significant interaction between EAT thickness and BAT activity status was observed (*p* = 0.919).

## 4. Discussion

In this single-center cross-sectional study of 207 adults evaluated for suspected MASLD, greater EAT thickness and lower IRT-derived ΔTemp were each associated with more severe CAP-defined hepatic steatosis. Both associations remained statistically significant after multivariable adjustment, with a stronger standardized association for EAT thickness. The EAT–CAP association remained consistent across lower and higher ΔTemp strata, with no statistically significant interaction. Collectively, these findings support EAT thickness and BAT-related thermogenic response as distinct yet complementary structural and functional adipose imaging phenotypes associated with hepatic steatosis. Their joint assessment offers an integrated imaging perspective on adipose-related heterogeneity in MASLD.

EAT is a metabolically active ectopic fat depot contiguous with the myocardium and coronary arteries. Through paracrine and vasocrine signaling, EAT has been linked to insulin resistance, chronic low-grade inflammation, and cardiometabolic dysfunction [21,25]. In the present study, EAT thickness increased with CAP-defined steatosis severity and remained independently associated with CAP after adjustment for BMI and metabolic variables; its standardized association with CAP was consistently larger than that of BMI across sequential models. The greater increase in EAT thickness among participants with moderate-to-severe steatosis further suggests that EAT expansion may become more evident as hepatic fat accumulates. These findings suggest that EAT thickness provides regional ectopic adiposity information distinct from that captured by BMI; however, whether the EAT–CAP association is independent of central or visceral adiposity remains uncertain. Consistent with previous studies, increased EAT has been associated with hepatic steatosis [26,27], while systematic evidence further supports its relevance to hepatic steatosis and disease severity [8,28]. From the perspective of the liver–heart metabolic axis, EAT may also help characterize the cardiometabolic implications of hepatic steatosis, given its associations with subclinical cardiovascular abnormalities and adverse cardiac remodeling [29,30,31]. Taken together, EAT thickness may represent a noninvasive imaging phenotype of regional ectopic adiposity associated with hepatic steatosis, with potential relevance to liver–cardiometabolic heterogeneity in MASLD.

In contrast to EAT, BAT represents a metabolically protective adipose phenotype characterized by UCP-1-mediated adaptive thermogenesis, energy expenditure, and regulation of systemic energy and hepatic lipid metabolism [10,32]. In the present study, BAT-related thermogenic activity was assessed using IRT-derived ΔTemp, which has been shown to correlate with ^18^F-FDG PET/CT-assessed BAT activity in previous validation studies [22,24]. We found that ΔTemp decreased with increasing CAP-defined steatosis severity and showed a modest independent inverse association with CAP after multivariable adjustment, including adjustment for EAT thickness. These findings are consistent with previous studies linking reduced BAT activity to steatotic liver disease and increased hepatic fat content [13,33]. Collectively, the modest inverse association between IRT-derived ΔTemp and CAP suggests that lower BAT-related thermogenic response may accompany more severe hepatic steatosis in MASLD, although the clinical relevance of this association warrants further investigation. This functional feature contrasts with the structural ectopic adiposity reflected by EAT thickness, providing a rationale for considering the two measures jointly.

The principal contribution of this study is the joint evaluation of two adipose-related imaging measures representing distinct domains: echocardiography-derived EAT thickness as a structural marker of regional ectopic adiposity and IRT-derived ΔTemp as an indirect thermographic marker of BAT-related thermogenic response. This distinction is consistent with prior imaging evidence that adipose depots may differ in their associations with metabolic and cardiac profiles [34]. When modeled together, EAT thickness and ΔTemp remained independently associated with CAP in opposite directions, with EAT thickness showing the stronger standardized association. This directionally opposite pattern suggests that more severe CAP-defined hepatic steatosis may be accompanied by both greater regional ectopic adiposity and lower BAT-related thermogenic response. In median-based stratified analyses, the EAT–CAP association remained consistent, with no evidence of effect modification by ΔTemp. Accordingly, EAT thickness and ΔTemp may be viewed as dual imaging phenotypes reflecting complementary structural and thermogenic adipose domains, despite their differing association strengths. Their joint assessment therefore offers an exploratory multimodal perspective on adipose-related heterogeneity in MASLD.

From a clinical imaging perspective, echocardiography-derived EAT thickness and IRT-derived ΔTemp can be obtained noninvasively and provide complementary structural and thermogenic adipose information. Echocardiography is widely available in routine practice, whereas IRT offers a radiation-free approach to estimating BAT-related thermogenic response. Considered alongside BMI and conventional metabolic variables, their joint assessment may provide a broader imaging perspective on adipose-related heterogeneity associated with hepatic steatosis. However, the clinical utility of this approach remains to be established through prospective validation.

Importantly, CAP, echocardiographic EAT thickness, and IRT-derived ΔTemp are indirect imaging biomarkers that reflect different aspects of hepatic and adipose biology. CAP estimates steatosis from hepatic ultrasound attenuation but is not equivalent to quantitative MRI-PDFF or histological assessment. Echocardiographic EAT thickness provides a one-dimensional measure of regional epicardial adiposity without characterizing total EAT volume, inflammation, or metabolic activity. Similarly, IRT-derived ΔTemp reflects a cold-induced skin-surface thermal response related to BAT thermogenesis and may be influenced by cutaneous perfusion, overlying tissue thickness, environmental conditions, and systemic physiological responses. Accordingly, these findings describe imaging-level relationships among hepatic steatosis, regional epicardial adiposity, and BAT-related thermogenic response, but do not directly establish the underlying tissue changes or biological mechanisms.

Several limitations should be acknowledged. First, the cross-sectional design is a major limitation and precludes determination of temporal sequence or causal relationships among EAT thickness, IRT-derived ΔTemp, and hepatic steatosis. Second, hepatic steatosis was assessed using CAP rather than MRI-PDFF or histology. Although validated for noninvasive steatosis assessment, CAP remains a surrogate measure with imperfect discrimination between adjacent grades. Third, IRT-derived ΔTemp is an indirect skin-surface marker of BAT-related thermogenic response rather than a direct measure of BAT volume or metabolic activity. Despite standardized room temperature and cold exposure, the potential effects of humidity, the timing and season of examination, cutaneous perfusion, and local skin and subcutaneous tissue thickness were not fully accounted for. Although no IRT examination was performed during active menstruation, menstrual-cycle phase and menopausal status were not otherwise systematically characterized. Fourth, although the sensitivity analysis additionally adjusted for diabetes and hypertension, direct measures of insulin resistance were unavailable, and detailed medication use was not systematically recorded. Residual confounding by these and other unmeasured factors cannot be excluded. Finally, the single-center design and modest sample size, particularly in the stratified analyses, may have limited both the ability to detect effect modification and the generalizability of the findings; external validation was not performed. Prospective multicenter studies incorporating quantitative liver fat assessment, BAT-sensitive imaging, and longitudinal follow-up are needed to validate these findings and clarify the directionality and clinical relevance of the observed associations.

## 5. Conclusions

In adults evaluated for suspected MASLD, echocardiographic EAT thickness and IRT-derived ΔTemp were independently associated with CAP-defined hepatic steatosis severity in opposite directions, with EAT thickness showing the stronger standardized association. These findings support their interpretation as dual imaging phenotypes for investigating adipose-related heterogeneity in MASLD and provide a basis for prospective longitudinal investigation.

## Figures and Tables

**Table 1:** Baseline characteristics of participants according to CAP-defined hepatic steatosis grades.

Variable	S0 (*n* = 45)	S1 (*n* = 57)	S2 (*n* = 46)	S3 (*n* = 59)	*p* Value
Age (years)	54 (38–60)	42 (30–55)	45 (32–54)	37 (32–44)	0.001
Male sex, *n* (%)	13 (28.9%)	25 (43.9%)	22 (47.8%)	42 (71.2%)	<0.001
BMI (kg/m^2^)	25.2 ± 3.3	26.6 ± 2.9	27.5 ± 3.8	28.9 ± 3.9	<0.001
CAP (dB/m)	224.2 ± 21.1	278.7 ± 13.2	313.6 ± 7.6	354.2 ± 20.9	<0.001
ALT (U/L)	49.0 (19.3–109.0)	73.0 (42.8–128.0)	51.0 (32.8–77.0)	71.5 (47.8–102.5)	0.005
AST (U/L)	41.5 (27.3–58.5)	56.0 (38.0–101.0)	38.0 (31.5–50.6)	44.0 (33.2–60.3)	0.002
TC (mmol/L)	4.87 (4.39–5.34)	5.03 (4.55–5.79)	5.24 (4.63–5.95)	5.26 (4.55–5.69)	0.393
TG (mmol/L)	1.36 (1.21–1.93)	1.50 (1.17–2.23)	1.66 (1.18–2.07)	1.74 (1.32–2.39)	0.259
LDL-C (mmol/L)	3.09 ± 1.09	3.20 ± 1.15	3.38 ± 1.06	3.21 ± 0.98	0.656
HDL-C (mmol/L)	1.18 (0.97–1.36)	1.07 (0.88–1.39)	1.00 (0.88–1.16)	1.02 (0.84–1.15)	0.019
EAT thickness (mm)	6.9 ± 1.6	7.2 ± 1.2	8.0 ± 1.0 ^ab^	10.0 ± 1.2 ^abc^	<0.001
ΔTemp (°C)	1.19 (0.78–1.87)	0.92 (0.76–1.22)	0.89 (0.77–1.29)	0.76 (0.58–0.94) ^abc^	<0.001

Table footnote: Data are presented as mean ± standard deviation (SD), median (interquartile range [IQR]), or *n* (%). Group comparisons were performed using one-way analysis of variance (ANOVA), Kruskal–Wallis test, or χ^2^ test, as appropriate. Post hoc pairwise comparisons were conducted using the Scheffé test for normally distributed variables and the Dunn–Bonferroni procedure for non-normally distributed variables. ^a^ *p* < 0.05 vs. S0 group; ^b^ *p* < 0.05 vs. S1 group; ^c^ *p* < 0.05 vs. S2 group. Abbreviations: BMI, body mass index; CAP, controlled attenuation parameter; ALT, alanine aminotransferase; AST, aspartate aminotransferase; TC, total cholesterol; TG, triglycerides; LDL-C, low-density lipoprotein cholesterol; HDL-C, high-density lipoprotein cholesterol; EAT, epicardial adipose tissue; ΔTemp, supraclavicular-to-chest temperature difference.

**Table 2:** Sequential multivariable linear regression models for factors associated with CAP.

Variables	Model 1	Model 2	Model 3	Model 4
EAT thickness (mm)	17.422 (0.597), <0.001	15.769 (0.623), <0.001	16.100 (0.586), <0.001	15.371 (0.559), <0.001
Male sex	14.033 (0.140), 0.020	11.341 (0.109), 0.047	11.934 (0.130), 0.048	13.368 (0.145), 0.024
Age (years)	−0.510 (−0.131), 0.027	−0.324 (−0.065), 0.143	−0.343 (−0.095), 0.156	−0.220 (−0.061), 0.359
BMI (kg/m^2^)		2.232 (0.201), 0.002	1.793 (0.143), 0.015	1.651 (0.132), 0.022
ALT (U/L)			0.001 (0.001), 0.995	0.020 (0.026), 0.652
TG (mmol/L)			3.393 (0.077), 0.187	3.615 (0.082), 0.151
HDL-C (mmol/L)			2.869 (0.027), 0.645	1.579 (0.015), 0.796
ΔTemp (°C)				−16.174 (−0.167), 0.004

Table footnote: Data are presented as unstandardized regression coefficients β (standardized β) and *p* values. CAP was included as the dependent variable in all models. Model 1 was adjusted for age and sex; Model 2 was additionally adjusted for BMI; Model 3 was additionally adjusted for ALT, TG, and HDL-C; and Model 4 was additionally adjusted for ΔTemp. Abbreviations: CAP, controlled attenuation parameter; EAT, epicardial adipose tissue; BMI, body mass index; ALT, alanine aminotransferase; TG, triglycerides; HDL-C, high-density lipoprotein cholesterol; ΔTemp, supraclavicular-to-chest temperature difference.

**Table 3:** Stratified multivariable linear regression analysis of CAP according to BAT activity status.

Variables	BAT-Low Activity Group (*n* = 103)	BAT-High Activity Group (*n* = 104)
EAT thickness (mm)	17.191 (0.662), <0.001	15.140 (0.511), <0.001
Male sex	19.641 (0.216), 0.004	5.569 (0.062), 0.578
Age (years)	0.605 (0.159), 0.041	−0.761 (−0.232), 0.047
BMI (kg/m^2^)	3.357 (0.275), <0.001	0.069 (0.006), 0.956
ALT (U/L)	0.124 (0.138), 0.043	−0.044 (−0.070), 0.499
TG (mmol/L)	1.852 (0.040), 0.551	3.752 (0.092), 0.338
HDL-C (mmol/L)	0.226 (0.002), 0.981	−0.429 (−0.005), 0.958
ΔTemp (°C)	−7.409 (−0.026), 0.694	−27.241 (−0.282), 0.006

Table footnote: Data are presented as unstandardized regression coefficients B (standardized β) and *p* values. Participants were stratified into BAT-low and BAT-high activity groups according to the median ΔTemp value. Separate multivariable linear regression models were constructed for each subgroup using CAP as the dependent variable. Abbreviations: CAP, controlled attenuation parameter; EAT, epicardial adipose tissue; BMI, body mass index; ALT, alanine aminotransferase; TG, triglycerides; HDL-C, high-density lipoprotein cholesterol; ΔTemp, supraclavicular-to-chest temperature difference.

## Data Availability

The data presented in this study are available from the corresponding authors upon reasonable request. The data are not publicly available due to participant privacy and ethical restrictions.

## Supplementary Materials

The following supporting information can be downloaded at https://www.mdpi.com/article/10.3390/diagnostics16172696/s1, Figure S1: Representative echocardiographic image for epicardial adipose tissue thickness measurement; Figure S2: Representative infrared thermographic image for assessment of BAT-related thermogenic activity; Table S1: Sensitivity analysis of the associations of EAT thickness and IRT-derived ΔTemp with CAP after additional adjustment for diabetes mellitus and hypertension.

## Abbreviations

The following abbreviations are used in this manuscript: ALT, alanine aminotransferase; AST, aspartate aminotransferase; BAT, brown adipose tissue; BMI, body mass index; CAP, controlled attenuation parameter; CI, confidence interval; EAT, epicardial adipose tissue; HDL-C, high-density lipoprotein cholesterol; IQR, interquartile range; IRT, infrared thermography; LDL-C, low-density lipoprotein cholesterol; MASLD, metabolic dysfunction-associated steatotic liver disease; MRI-PDFF, magnetic resonance imaging–proton density fat fraction; ^18^F-FDG PET/CT, fluorine-18 fluorodeoxyglucose positron emission tomography/computed tomography; ROI, region of interest; RV, right ventricle; TC, total cholesterol; Tc, chest reference temperature; TE, transient elastography; TG, triglyceride; Tscv, supraclavicular skin temperature; UCP-1, uncoupling protein-1.
