# Supplementary material for: EAT Thickness and BAT-Related Thermogenesis: Dual Imaging Phenotypes Associated with Hepatic Steatosis Severity in MASLD
Source: Diagnostics (Basel). 2026 Aug 24;16(17):2696. doi: 10.3390/diagnostics16172696 (PMC13564783; doi:10.3390/diagnostics16172696)
Supplement: Supplementary file 1 [file diagnostics-16-02696-s001.zip › diagnostics-4440989-supplementary.pdf]

Supplementary Materials

Figure S1. Representative echocardiographic image for epicardial adipose tissue thickness measurement.

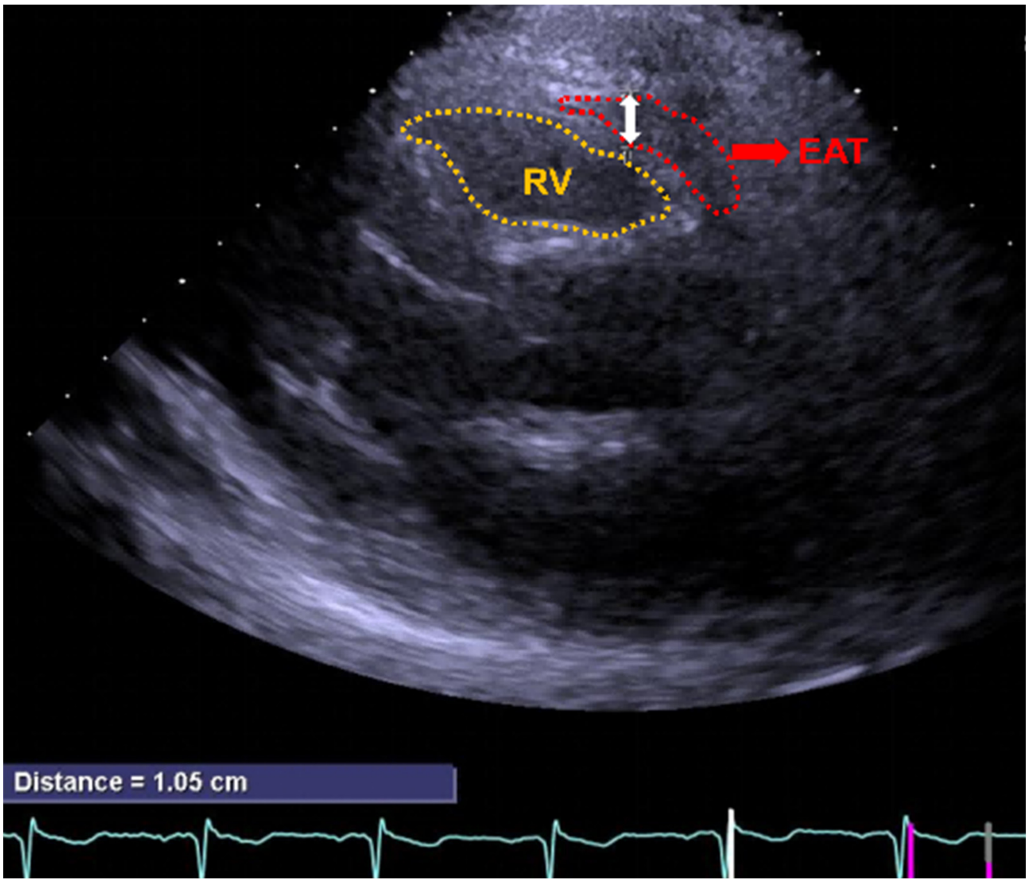

Representative parasternal long-axis transthoracic echocardiographic image showing epicardial adipose tissue (EAT) thickness measurement. The EAT depot (red outline) was identified as the echo-free space between the outer wall of the myocardium and the visceral pericardium. The right ventricle (RV; yellow outline) is shown as an anatomical reference. EAT thickness was measured perpendicularly over the RV free wall at end-systole (white double-headed arrow). The mean value from three consecutive cardiac cycles was used for analysis.

Abbreviations: EAT, epicardial adipose tissue; RV, right ventricle.

Figure S2. Representative infrared thermographic image for assessment of BAT-related thermogenic activity.

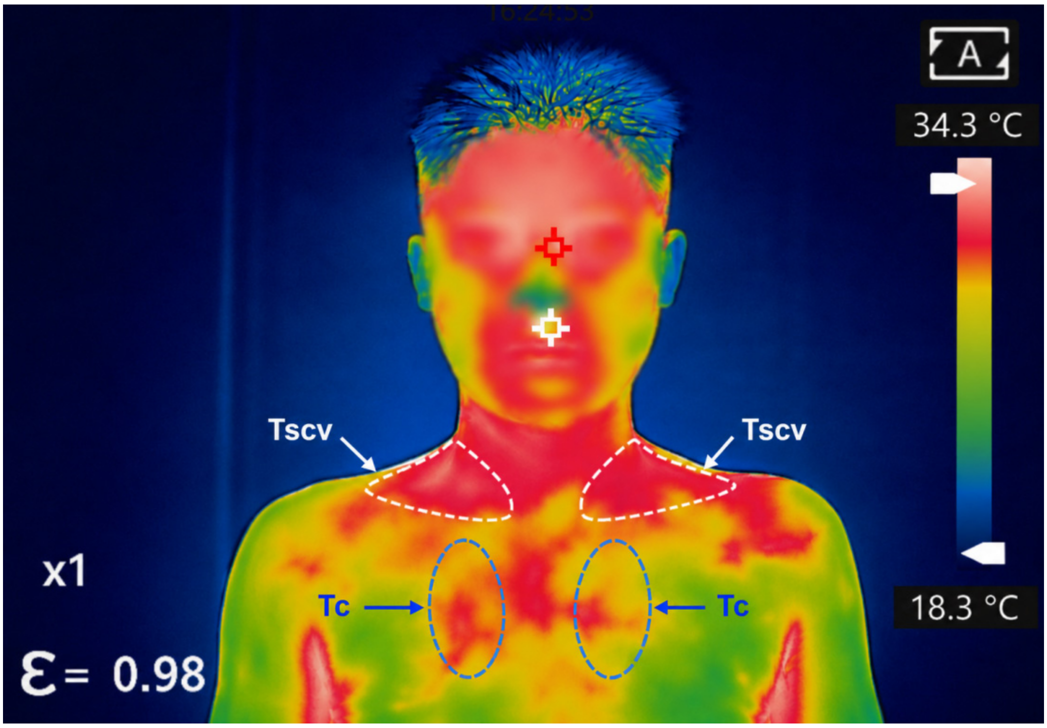

Representative infrared thermographic image obtained after 30 min of mild cold exposure (19°C). White dashed outlines indicate the bilateral supraclavicular regions of interest (ROIs) corresponding to brown adipose tissue (BAT) depots, where the highest temperature was recorded as the supraclavicular skin temperature (Tscv). Blue dashed outlines indicate the bilateral parasternal upper chest reference ROIs, where the mean temperature was recorded as the chest reference temperature (Tc). BAT-related thermogenic activity was quantified as the temperature difference between the supraclavicular and reference regions (ΔTemp = Tscv − Tc).

Abbreviations: BAT, brown adipose tissue; ROI, region of interest; Tscv, supraclavicular skin temperature; Tc, chest reference temperature.

Table S1. Sensitivity analysis of the associations of EAT thickness and IRT-derived ΔTemp with CAP after additional adjustment for diabetes mellitus and hypertension

Variable	B (95% CI)	Standardized β	*P* value	Tolerance	VIF
EAT thickness (mm)	14.862 (11.803 to 17.921)	0.551	<0.001	0.901	1.109
Male Sex	14.109 (2.296 to 25.922)	0.154	0.020	0.698	1.432
Age (years)	-0.251 (-0.744 to 0.243)	-0.070	0.317	0.612	1.635
BMI (kg/m²)	1.547 (0.124 to 2.970)	0.124	0.033	0.890	1.124
ALT (U/L)	0.015 (-0.072 to 0.102)	0.021	0.727	0.853	1.172
TG (mmol/L)	3.559 (-1.472 to 8.590)	0.081	0.164	0.888	1.127
HDL-C (mmol/L)	1.794 (-10.281 to 13.869)	0.017	0.770	0.885	1.130
ΔTemp (°C)	-16.017 (-26.923 to -5.111)	-0.166	0.004	0.911	1.097
Hypertension	3.675 (-8.909 to 16.259)	0.034	0.565	0.854	1.171
Diabetes mellitus	5.180 (-7.845 to 18.205)	0.045	0.433	0.894	1.119

Footnote: CAP was the dependent variable. The sensitivity model corresponded to Model 4 with additional adjustment for diabetes mellitus and hypertension. B represents the unstandardized regression coefficient, and β represents the standardized regression coefficient.

Abbreviations: ALT, alanine aminotransferase; BMI, body mass index; CAP, controlled attenuation parameter; CI, confidence interval; EAT, epicardial adipose tissue; HDL-C, high-density lipoprotein cholesterol; IRT, infrared thermography; TG, triglycerides; VIF, variance inflation factor; ΔTemp, supraclavicular-to-chest temperature difference.
